# A Polymorphism in the Chitotriosidase Gene Associated with Risk of Mycetoma Due to *Madurella mycetomatis* Mycetoma–A Retrospective Study

**DOI:** 10.1371/journal.pntd.0004061

**Published:** 2015-09-02

**Authors:** Patricia E. B. Verwer, Charlotte C. Notenboom, Kimberly Eadie, Ahmed H. Fahal, Henri A. Verbrugh, Wendy W. J. van de Sande

**Affiliations:** 1 Dept. of Medical Microbiology & Infectious Diseases, Erasmus MC, University Medical Centre Rotterdam, CE Rotterdam, The Netherlands; 2 Mycetoma Research Centre, Khartoum, Sudan; University of Tennessee, UNITED STATES

## Abstract

**Background:**

*Madurella mycetomatis* is the most prevalent causative agent of eumycetoma in Sudan, an infection characterized by the formation of grains. Many patients are exposed to the causative agent, however only a small number develop infection. *M*. *mycetomatis* contains chitin in its cell wall, which can trigger the human immune system. Polymorphisms in the genes encoding for the chitin-degrading enzymes chitotriosidase and AMCase were described, resulting in altered chitinase activity. We investigated the association between 4 of these polymorphisms and the incidence of *M*. *mycetomatis* mycetoma in a Sudanese population.

**Methodology:**

Polymorphisms studied in 112 eumycetoma patients and 103 matched controls included a 24-bp insertion in the chitotriosidase gene (rs3831317), resulting in impaired chitinase activity and single nucleotide polymorphism (SNP) in the AMCase gene (rs61756687), resulting in decreased AMCase activity. Also, a SNP (rs41282492) and a 10-bp insertion in the 5’UTR region of the AMCase gene (rs143789088) were studied, both resulting in increased AMCase activity. DNA was isolated from blood and genotypes were determined using PCR-RFLP.

**Principal Findings:**

Histological staining proved the presence of chitin in the fungal grain. The polymorphism resulting in decreased chitotriosidase activity was associated with increased odds of eumycetoma (odds ratio 2.9; p = 0.004). No association was found for the polymorphisms in the genes for AMCase (all p>0.05).

**Conclusion:**

Decreased chitotriosidase activity was associated with increased risk of *M*. *mycetomatis* mycetoma.

## Author Summary

*Madurella mycetomatis* mycetoma is a chronic fungal infection, resulting frequently in mutilating lesions. The causative agents are found in soil, however, many people are exposed but most do not develop mycetoma. Characteristic for mycetoma is that the fungus organizes itself in a grain once inside the body. Here we showed that this grain contains chitin. The immune system of the host will try to eliminate the grains by producing cytokines and enzymes, including the chitin-degrading chitinases. We showed that both human chitinases AMCase and chitotriosidase bind to fungal chitin in the grain. We also investigated 4 polymorphisms in the genes for these chitinases, and we found that a polymorphism in the gene for chitotriosidase, resulting in enzyme inactivity, was associated with increased risk for mycetoma. Based on these findings, we hypothesized that chitotriosidase is important in the pathogen-eliminating immune response, resulting in clearance of the infection. We assumed that absence of chitotriosidase results in increased AMCase production and thus in grain formation. In this study we identified a risk factor for the development of *M*. *mycetomatis* mycetoma, however the disease is multifactorial and other factors also play a role.

## Introduction

Mycetoma is a chronic infectious granulomatous disease that is frequently reported in tropical and subtropical climates between 30°N and 15°S of the equator. Mycetoma can be caused by bacteria (actinomycetoma) and fungi (eumycetoma), though the fungus *Madurella mycetomatis* is the most common causative agent in the world [1,2]. In Sudan it accounts for over 70% of all mycetoma cases [1]. The lower extremities are affected most, though other sites of the body can be affected as well [1]. After traumatic inoculation of the causative agent into the subcutaneous tissue, usually in the sole of the foot, the disease progresses and invades the deep structures and the skin. Multiple nodules and sinuses discharging pus are formed and grains develop. In the grains, the fungal mycelium is embedded in hard brown matrix containing melanin. This grain is thought to protect the fungus from the host immune system.

In endemic areas in Sudan, *M*. *mycetomatis* DNA was found in the soil [3] and antibodies against mycetoma causative agents have been detected in the majority of the inhabitants in these areas [4]. However, it is not clear why only a minority of the exposed humans develop overt clinical infection. Several environmental and patient-related factors have been described to influence the risk for the development of mycetoma, including concurrent schistosomiasis [5]. Furthermore, several associations with genetic polymorphisms in genes involved in hormone synthesis [6] and parts of the immune system, including collagenases and gelatinases were reported [4, 7, 8]. However, the role of chitinases in eumycetoma caused by *M*. *mycetomatis* was not investigated previously.

Infection with *M*. *mycetomatis* often results in fungal grain development in the tissue. The exact composition of the grain is not known. Ibrahim *et al* reported that the grains contain melanin, heavy metals, proteins and lipids, resulting in a cement matrix in which the mycelium is embedded [9]. Probably, the grain also contains chitin, since chitin is one of the cell wall components of many fungi. Around the fungal grain, the host’s innate immune system mounts an inflammatory response, resulting in a reactive granuloma laden with neutrophils, macrophages and other inflammatory cells [10]. Chitinases are part of the innate immune system and, in general, their production by macrophages and other cells is upregulated by the host’s tissue exposure to chitin. Chitinases seem to play a role in allergic and infectious diseases caused by chitin-bearing organisms [2, 11, 12], however the exact role of these enzymes in pathogenesis is yet to be elucidated. Chitinases hydrolyze chitin, which is the main component of the fungal cell wall. Decreased chitinase activity could, therefore, result in an enhanced susceptibility towards infections by chitin bearing fungi. Currently, two true chitinases are known in humans: chitotriosidase and acidic mammalian chitinase (AMCase)[13]. Polymorphisms in the genes for these chitinases have been described that are associated with either increased or decreased enzyme activity [12,14–17]. Impaired or decreased activity of either one or both of these chitinases could result in increased susceptibility towards fungal infections including eumycetoma. The aim of this study is to investigate the role of chitinase activity in the development of mycetoma caused by *M*. *mycetomatis*. We hypothesized that chitotriosidase and AMCase polymorphisms, resulting in decreased chitinase activity, would be found more frequently in mycetoma patients than in controls.

## Methods

### Study population

Individuals presenting at Mycetoma Research Center, Khartoum between 2001 and 2008, were eligible for inclusion, when the diagnosis of *Madurella mycetomatis* mycetoma was confirmed. People living in the same endemic regions were included as controls. The demographic features are given in Table 1. Retrospectively, genotypes were determined of 112 *Madurella mycetomatis* infected patients and 103 healthy endemic controls, matched for sex and age. Whole blood was stored at -80°C until processing. DNA was isolated from whole blood of these subjects using the MagNA Pure LC DNA Isolation kit—Large Volume (Roche Diagnostics Nederland BV, Almere, the Netherlands) according to the manufacturer’s instructions. DNA was stored at -20°C until processing. Tissue of the foot and of the grain was obtained in 1998 from Sudanese subjects, infected with *M*. *mycetomatis*.

**Table 1:** Study population demographic features.

Characteristic		Mycetoma patients (n = 112)	Endemic controls (n = 103)
**Gender (male/female)**		79/33	77/26
**Mean duration in years (range)**		6.9 (1–27)	N/A
**Mycetoma lesion site** *	Foot	87	N/A
	Hand	12	N/A
	Lower leg	14	N/A
**Mycetoma lesion size**	Small (<5 cm)	55	N/A
	Moderate (5–10 cm)	20	N/A
	Massive (>10 cm)	38	N/A

* One patient had two lesions, one of the foot and one of the hand

### Genotyping

PubMed Library was searched for publications describing polymorphisms in the chitotriosidase gene and in the AMCase gene. We included only polymorphisms that were described elsewhere to result in an alteration (either increase or decrease) of the chitinase enzymatic activity. PCR primers, amplification conditions and restriction enzymes used in this study are shown in Table 2. We purchased all enzymes at Fermentas (Thermo Fisher Scientific, Waltham, USA). Enzymes were used according to manufacturer’s guidelines. We determined genotypes by polymerase chain reaction restricted fragment length polymorphism (PCR-RFLP). PCR products were run at a 2.5% metaphor gel in 90 minutes. Two different investigators assessed genotypes separately.

**Table 2:** PCR conditions for the different polymorphisms.

Polymorphism	Primer sequence (5’-> 3’)	PCR program	Restriction endonuclease	Activity	Length (bp)	Ref
Chitotriosidase 24-bp insertion	F: agctatctgaagcagaag	4’ 94°C + 40x (30” 94°C + 30”	None	Normal	124 bp	[14, 19, 20]
rs 3831317	R: ggagaagccggcaaagtc	55°C + 30” 72°C) + 7’ 72°C		Decreased	148 bp	
AMCase A50G	F: gtctcaccctgccttctttg	4’ 94°C + 40x (30” 94°C + 30”	ApoI (XapI)	Normal	175 + 91	[21]
rs 61756687	R: acccaattctcctcggaaag	58°C + 30” 72°C) + 7’ 72°C		Decreased	266	
AMCase A290G	F: ctctgcctaccagctgacat	4’ 94°C + 40x (30” 94°C + 30”	TaqI	Normal	256 + 81 + 69	[17]
rs 41282492	R: gccattccgcaccgtataca	58°C + 30” 72°C) + 7’ 72°C		Increased	256 + 150	
AMCase 10-bp insertion 5’UTR	F: ctgaccacagtatctaaacag	4’ 94°C + 40x (30” 94°C + 30”	BfaI	Normal	392 + 59	[16]
rs 143789088	R: ctgaccacagtatctaaacag	58°C + 30” 72°C) + 7’ 72°C		Increased	308 + 94 + 59	

### Immunohistochemistry

Tissue biopsies were fixed in formalin, embedded in paraffin and processed for immunohistochemical evaluation. To determine if the antibodies directed against human AMCase and chitotriosidase did not cross-react with any fungal proteins, heat-fixed *in vitro* grown *M*. *mycetomatis* hyphae were stained with the same protocol. Histologic sections (coupes) were deparaffinised in xylene, then rehydrated in decreasing concentrations of ethanol. Haematoxylin and eosin staining was used as standard staining. Calcofluor-white staining was used to visualize chitin, according to manufacturer’s guidelines. Coupes were washed in aquadest and incubated with 25 μM calcofluor-white (Molecular Probes, Leiden, The Netherlands) for 30 minutes at 37°C in the dark. Afterwards, coupes were washed in aquadest and assessed by fluorescent microscopy.

Presence of chitinase was determined as described previously [2]. First, endogenous peroxidase was blocked in methanol with 0.3% H_2_O_2_ and non-specific binding sites were blocked with rabbit or goat serum. Subsequently, coupes were incubated overnight with rabbit polyclonal antibody directed against chitotriosidase (H-66, 1:75, Santa Cruz Biotechnology, Santa Cruz, USA) or with goat polyclonal antibody directed against AMCase (Y-14, 1:50, both Santa Cruz Biotechnology, Santa Cruz, USA). As a control, a goat polyclonal IgG antibody was used, directed against swine IgM (A100-100A, 1:50, Bethyl Laboratories, Montgomery, USA). From the VectaStain® Elite ABC kit (Vector Laboratories Burlingame, CA, USA), we used anti-rabbit IgG or anti-goat IgG as a secondary antibody and the coupes were developed using the protocol from the kit. Haematoxylin was used as counter staining.

### Ethics statement

Written informed consent was obtained from patients and controls, according to guidelines from the medical ethical committee at Soba University Hospital, Khartoum, Sudan. The study protocol was approved by this medical ethical committee.

### Statistical analysis

Pearson’s χ^2^ test was used to verify the Hardy-Weinberg equilibrium. Differences in allele frequencies were determined using the two-sided Fisher’s exact test (GraphPad Prism Software, San Diego, USA). A p-value of p < 0.05 was considered significant.

## Results

### Mycetoma grains caused by *Madurella mycetomatis* contain chitin and chitinase

**Fig 1:**
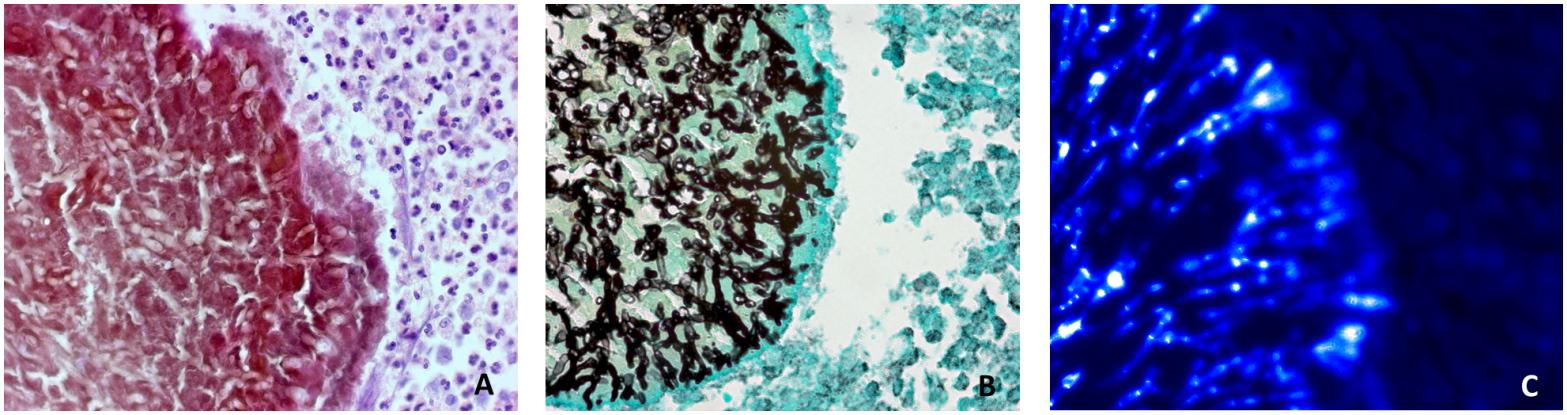
Tissue sections of Mycetoma foot, showing the fungal grain. Magnification 400x. (A) Haematoxylin and eosin staining. The grain, consisting of cement and fungal hyphae, is colored red. Around the grain, a zone with neutrophils is visible. (B) Grocott’s methenamine silver staining. The hyphae inside the grain are stained black. (C) Calcofluor white staining. Chitin is stained by calcofluor white staining [18]. This photo illustrates that hyphae inside the grain are stained, and not the cement component of the grain.

**Fig 2:**
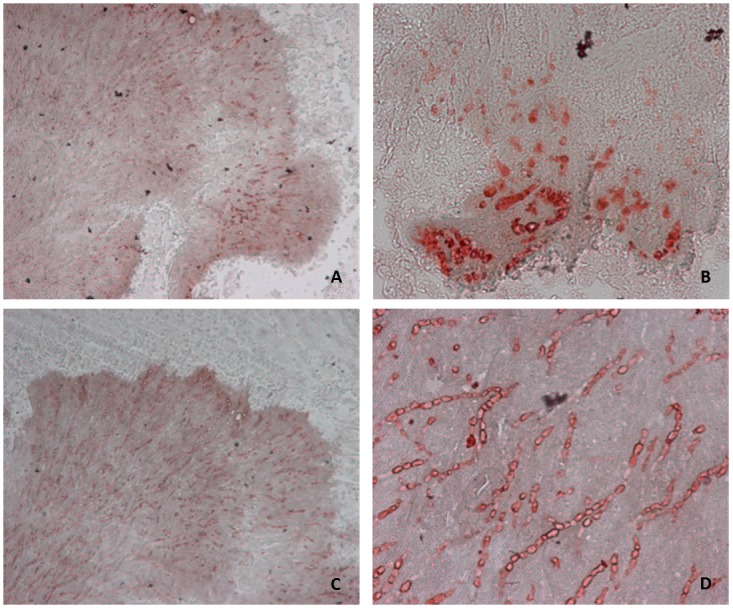
Tissue sections of Mycetoma foot stained for chitotriosidase and for AMCase. Magnification 100x (A and C) and 400x (B and D). (A) and (B): Chitotriosidase. (C) and (D): AMCase. Presence of both enzymes is shown by red color. The grain is clearly visible and colored red diffusely. Inside the grain, fungal hyphae are stained more intensely, showing an increased presence of chitotriosidase and AMCase around the fungal hyphae.

Tissue sections of the fungal grain caused by *M*. *mycetomatis* are shown in Fig 1. The sections were stained using haematoxylin and eosin (HE) (Fig 1A), grocott’s methenamine silver stain (Fig 1B) and calcofluor-white stain (Fig 1C). In Fig 1A it is clearly seen that the grain itself contains cement material. The hyphae are embedded within this cement material (Fig 1B). In Fig 1C, fungal chitin is stained by calcofluor-white [18]. This figure shows that the chitin is mainly found in the hyphae inside the grain. The cement material itself is not stained. Since chitin is present in the hyphae inside the grain, we hypothesized that the host produces chitinases, in reaction to exposure to chitin. Thus, tissue sections were stained for chitotriosidase and for AMCase (Fig 2). The presence of chitinases is shown by a red color, which was mainly located in the grain on the fungal hyphae and not in the cement component of the grain. This seems consistent with the presence of the chitin itself, which was also mainly found on the fungal hyphae and not in the cement material. We also found that both chitotriosidase and AMCase were found in the tissue surrounding the fungal grain, however both chitinases concentrated in the grain. The staining reaction for chitotriosidase was specific, since no binding of the chitotriosidase specific antibodies was noted on *in vitro* grown fixated *M*. *mycetomatis* hyphae. In contrast, binding of the AMCase specific antibodies was noted on some *in vitro* grown, fixated *M*. *mycetomatis* hyphae, although the staining was less intense than that inside the grain. Therefore it is plausible that not only chitotriosidase but also AMCase are indeed binding to the hyphae inside the mycetoma grain.

### Polymorphisms in the genes for chitinases cause a risk for infection with *M*. *mycetomatis*

We subsequently investigated whether genetic polymorphisms that resulted in either increased or decreased activity of either chitinase, were associated with an increased risk for invasive fungal mycetoma disease. Exactly 215 Sudanese subjects were included in the study and were genotyped. Of these, 112 subjects had active mycetoma disease and were classified as patients, and 103 subjects were healthy controls. Genotype frequencies are shown in Table 3. The genotype distribution for all polymorphisms was consistent with the Hardy Weinberg equilibrium.

**Table 3:** Distribution of polymorphisms in the genes for chitotriosidase and for AMCase.

Gene Polymorphism	Genotype	Enzyme activity*	Patients (%) n = 112	Controls (%) n = 103	HWE** p-value	p-value	Odds ratio (95% CI interval)
Chitotriosidase 24-bp insertion	Wildtype	Normal	84 (75%)	94 (91%)	0.106	0.004	2.9 (1.4–6.1)
	Heterozygous 24-bp insertion	Decreased	27 (24%)	8 (8%)			
	Homozygous 24-bp insertion	Impaired	1 (1%)	1 (1%)			
AMCase A50G	AA	Normal	92 (82%)	83 (81%)	0.940	0.647	1.1 (0.7–1.8)
	AG	Normal	14 (13%)	19 (18%)			
	GG	Decreased	6 (5%)	1 (1%)			
AMCase A290G	AA	Normal	74 (66%)	67 (65%)	0.657	0.717	1.2 (0.6–2.1)
	AG	Normal	30 (27%)	33 (32%)			
	GG	Increased	8 (7%)	3 (3%)			
AMCase 10-bp insertion 5’UTR	Wildtype	Normal	73 (65%)	66 (64%)	0.578	0.720	1.1 (0.7–1.8)
	Heterozygous 10-bp insertion	Normal	31 (28%)	34 (33%)			
	Homozygous 10-bp insertion	Increased	8 (7%)	3 (3%)			

*Genotype associated with an impaired, normal or decreased enzyme activity according to previous publications [14, 16, 17, 19–21]

**Hardy Weinberg Equilibrium (HWE) as assessed by Pearson’s χ^2^ test. A p-value of >0.05 was associated with equilibrium.

### The 24-bp insertion in the chitotriosidase gene is associated with invasive mycetoma

In the gene encoding for chitotriosidase, a 24-bp insertion in exon 10 was described previously [12, 14, 19, 20]. It was described elsewhere that chitotriosidase activity is reduced when patients are heterozygous for this insertion and completely absent when they were homozygous for this allele [12, 14]. Among the patients, 1/112 (0.9%) was homozygous for the 24-bp insertion and 27/112 (24.1%) were heterozygous. Among the controls, 1/103 (1.0%) was homozygous for the insertion and 8/103 (7.8%) were heterozygous. The insertion containing allele was found significantly more frequently in the patients than in the control group (p = 0.004). Based on these data, the 24-bp insertion in the chitotriosidase gene does increase the risk for invasive mycetoma disease caused by *M*. *mycetomatis* (odds ratio 2.9; 95% CI 1.4–6.1).

### Neither the A50G and the A290G SNP, nor the 10-bp insertion in the 5’UTR region of AMCase is associated with invasive mycetoma

Several SNPs in the AMCase genes were described previously, resulting in either increased or decreased AMCase activity [16, 17, 21]. As shown in table 2, we studied two SNPs (A50G and A290G) and one 10-bp insertion in the 5’UTR region of the AMCase gene. Allele frequencies of A50G and A290G in the patients were similar to those in the controls (p = 0.65; odds ratio 1.2; 95% CI 0.6–2.1; and p = 0.72; odds ratio 1.1; 95% CI 0.7–1.8, respectively). Furthermore, no difference was found in the presence of the 10-bp insertion between patients and controls (p = 0.72; odds ratio 1.1; 95% CI 0.7–1.8).

## Discussion

*Madurella mycetomatis* is the most prevalent causative agent of eumycetoma worldwide and in Sudan in particular [1]. Many inhabitants of Sudan are exposed to this causative agent, however few of them develop mycetoma. Currently, the predisposing factors for mycetoma are not known, but some genetic polymorphisms have been associated with the development of mycetoma [4, 6, 7].

In this paper, we first showed that chitin is present in the *M*. *mycetomatis* grain and that two human chitinases are found in the vicinity of this grain, in reaction to exposure to *M*. *mycetomatis* mycetoma. Both AMCase and chitotriosidase seemed to concentrate on the chitin-containing fungal hyphae, a phenomenon that was previously found in rats infected with *Aspergillus fumigatus* [2]. Since in both mycetoma and in *A*. *fumigatus* infected tissue AMCase and chitotriosidase concentrated on chitin-containing fungal hyphae, it needed to be determined if this was not due to cross-reaction of fungal proteins with the antibodies used. Staining *in vitro* grown *M*. *mycetomatis* hyphae with an antibody for chitotriosidase showed no staining on the hyphae. Also, no staining of *A*. *fumigatus* hyphae occurred [2], making it likely that the chitotriosidase antibodies were specifically directed against mammalian chitotriosidase and that fungi expressed no proteins which share epitopes with this enzyme. However, it should be kept in mind that *in vitro* grown fungi could express different proteins than could be expressed in a grain. In contrast to the specificity of the chitotriosidase antibody, the AMCase antibody seemed less specific. When stained with the AMCase antibody, some staining of the *in vitro* grown *M*. *mycetomatis* hyphae was noted. This was not the case for *A*. *fumigatus* hyphae [2]. Apparently, a protein with an epitope similar to that of AMCase is located on some fungal hyphae when grown *in vitro*. Therefore the AMCase stained in the tissue samples of the patients could be the result of both expressed human AMCase and a protein of fungal origin. Since the staining was more intense in the tissue sections, we feel that it is likely that AMCase was indeed present.

Next to demonstrating that AMCase and chitotriosidase were present at the site of infection, we also provided evidence that a polymorphism in the gene for chitotriosidase, resulting in impaired enzyme activity, significantly increased the risk for the development of eumycetoma. Increased or decreased activity of the alternative human chitinase, AMCase, did not have a significant influence on the risk for eumycetoma. In *M*. *mycetomatis* mycetoma, chitotriosidase is apparently more crucial than AMCase. Although chitotriosidase and AMCase are both chitinases, the cleavage site of both chitinases differs. Chitotriosidase is an exochitinase, whereas AMCase is an endochitinase, referring to the site where the enzyme cleaves the chitin chain [22]. Apparently, exochitinase activity is more important in the prevention of mycetoma than endochitinase activity. This is supported by the fact that in certain diseases an association was found with only one chitinase and not with both. In genetic association studies conducted in patients with bronchial asthma, an association was reported with only one chitinase, and not with both of them [21, 23, 24], indicating that both chitinases may have the same substrate, but have a distinct function in humans. Chitin and the produced chitotriosidase seem to be important in the development of the mycetoma grain. Since many mycetoma patients have the wild type chitinases associated with normal levels and activity of these enzymes, however, polymorphisms in these enzyme-encoding genes are clearly not the only factors that determine the risk for *M*. *mycetomatis* mycetoma.

Many previous reports showed that polarization of the immune response seems to play a role in the development of mycetoma [4, 7, 25–28]. The development of mycetoma is associated with a Th2 response. Based on immunohistochemistry studies in various mycetoma causative agents, it appeared that the cytokine pattern surrounding the mycetoma grain is a Th2 response. IL-10 and IL-4, both Th2-associated cytokines, were highly expressed around the fungal grain [7, 26, 27]. A different study showed that after stimulation of peripheral blood mononuclear cells (PBMCs) with mycetoma antigens, a Th2 response developed in mycetoma patients and a Th1 response developed in healthy endemic controls [25]. Indirect evidence for a Th2 response was also found in the association between schistosomiasis, associated with a Th2 response, and eumycetoma [5]. Not only cytokines, but also other mediators matching Th2 response were reported in mycetoma.

Sandler *et al* [28] showed that mice with a Th2 response have increased expression of matrix metalloproteinases (MMPs) and of tissue inhibitor of MMP-1 (TIMP-1). Furthermore, AMCase was induced in Th2-polarized mice [28], which was confirmed by several other studies [29–31]. Furthermore, Geneuglijk *et al* confirmed that MMP-2 and MMP-9 were expressed in the mycetoma lesion [8]. In this paper we also demonstrated that AMCase and chitotriosidase are expressed in the mycetoma lesion.

In contrast to AMCase, which is induced in a Th2 response, chitotriosidase is produced in the environment of a Th1 response [32]. In our study we showed that impaired function of chitotriosidase increases the risk to develop mycetoma.

Since Elagab et al already demonstrated that the PBMCs of healthy endemic controls produce Th1 cytokines when exposed to *M*. *mycetomatis* antigens, it is likely that they also produce high levels of chitotriosidase in order to eliminate *M*. *mycetomatis*. In individuals with a genotype resulting in impaired chitotriosidase activity, elimination of *M*. *mycetomatis* could be less efficient, leading to the development of a mycetoma lesion. More research is needed to unravel the exact role of the host in the development of the mycetoma grain.

Based on the data of this study and of other studies, we created the following hypothesis. When a host is exposed to *M*. *mycetomatis*, it will try to eliminate the pathogen. Most individuals will respond with a Th1 polarized response. In this response acute inflammation develops, chitotriosidase is produced and no grain is formed. When the response is mainly Th2-polarized, a chronic inflammatory process in which both chitotriosidase and AMCase are produced, results in the formation of granulomas with grains. The chronic exposure to fungal material in combination with the Th2 response also results in an increase in MMPs, which further modulates the collagen capsule surrounding the grain [2, 8, 28]. Polymorphisms in the genes for IL-10, CCL-5 and TIMP-1 were shown to increase the risk for mycetoma [7, 8]. Chitinases degrade the chitin component of the fungal cell wall [2]. A polymorphism in the gene for chitotriosidase results in impaired enzyme activity [14], which in its turn results in an increased risk of developing *Madurella mycetomatis* mycetoma, as we showed in this paper.

In conclusion, in this study we demonstrated that the grain caused by *Madurella mycetomatis*, contains chitin. The human immune system produced both AMCase and chitotriosidase in the vicinity of this grain. Only the 24-bp insertion in the gene for chitotriosidase was associated with the development of mycetoma caused by *M*. *mycetomatis*.

## Supporting Information

S1 Checklist STROBE checklist. (DOCX)
