# Supplementary material for: A Polymorphism in the Chitotriosidase Gene Associated with Risk of Mycetoma Due to Madurella mycetomatis Mycetoma–A Retrospective Study
Source: PLoS Negl Trop Dis. 2015 Sep 2;9(9):e0004061. doi: 10.1371/journal.pntd.0004061 (PMC4558086; doi:10.1371/journal.pntd.0004061)
Supplement: S1 Checklist — (DOCX) [file pntd.0004061.s001.docx]

STROBE Statement—checklist of items that should be included in reports of observational studies

|  | Item No. | Recommendation | Page  No. | Relevant text from manuscript |
| --- | --- | --- | --- | --- |
| **Title and abstract** | 1 | (*a*) Indicate the study’s design with a commonly used term in the title or the abstract | 1 | A retrospective study |
|  |  | (*b*) Provide in the abstract an informative and balanced summary of what was done and what was found | 2 | We investigated the association between 4 of these polymorphisms and the incidence of *M. mycetomatis* mycetoma in a Sudanese population.  Methodology Polymorphisms studied in 112 eumycetoma patients and 103 matched controls included a 24-bp insertion in the chitotriosidase gene (rs3831317), resulting in impaired chitinase activity and single nucleotide polymorphism (SNP) in the AMCase gene (rs61756687), resulting in decreased AMCase activity. Also, a SNP (rs41282492) and a 10-bp insertion in the 5’UTR region of the AMCase gene (rs143789088) were studied, both resulting in increased AMCase activity. DNA was isolated from blood and genotypes were determined using PCR-RFLP.  Principal findings Histological staining proved the presence of chitin in the fungal grain. The polymorphism resulting in decreased chitotriosidase activity was associated with increased odds of eumycetoma (odds ratio 2.9; p=0.004). No association was found for the polymorphisms in the genes for AMCase (all p>0.05). |
| Introduction | | | |  |
| Background/rationale | 2 | Explain the scientific background and rationale for the investigation being reported | 4 | In endemic areas in Sudan, *M. mycetomatis* DNA was found in the soil [3] and antibodies against mycetoma causative agents have been detected in the majority of the inhabitants in these areas [4]. However, it is not clear why only a minority of the exposed humans develop overt clinical infection. Several environmental and patient-related factors have been described to influence the risk for the development of mycetoma, including concurrent schistosomiasis [5]. Furthermore, several associations with genetic polymorphisms in genes involved in hormone synthesis [6] and parts of the immune system, including collagenases and gelatinases were reported [4, 7, 8]. |
| Objectives | 3 | State specific objectives, including any prespecified hypotheses | 5 | The aim of this study is to investigate the role of chitinase activity in the development of mycetoma caused by *M. mycetomatis*. We hypothesized that chitotriosidase and AMCase polymorphisms, resulting in decreased chitinase activity, would be found more frequently in mycetoma patients than in controls. |
| Methods | | | |  |
| Study design | 4 | Present key elements of study design early in the paper | 6  6 | Individuals presenting at Mycetoma Research Center, Khartoum between 2001 and 2008, were eligible for inclusion, when the diagnosis of *Madurella mycetomatis* mycetoma was confirmed. People living in the same endemic regions were included as controls.  Retrospectively, genotypes were determined of 112 *Madurella mycetomatis* infected patients and 103 healthy endemic controls, matched for sex and age. |
| Setting | 5 | Describe the setting, locations, and relevant dates, including periods of recruitment, exposure, follow-up, and data collection | 6 | See above, item 4. |
| Participants | 6 | (*a*) *Cohort study*—Give the eligibility criteria, and the sources and methods of selection of participants. Describe methods of follow-up  *Case-control study*—Give the eligibility criteria, and the sources and methods of case ascertainment and control selection. Give the rationale for the choice of cases and controls  *Cross-sectional study*—Give the eligibility criteria, and the sources and methods of selection of participants | 6 | See above, item 4. |
|  |  | (*b*) *Cohort study*—For matched studies, give matching criteria and number of exposed and unexposed  *Case-control study*—For matched studies, give matching criteria and the number of controls per case | - | Individuals were not matched. |
| Variables | 7 | Clearly define all outcomes, exposures, predictors, potential confounders, and effect modifiers. Give diagnostic criteria, if applicable | - | Outcome is epidemiological data. |
| Data sources/ measurement | 8* | For each variable of interest, give sources of data and details of methods of assessment (measurement). Describe comparability of assessment methods if there is more than one group | 6 | Two different investigators assessed genotypes separately. |
| Bias | 9 | Describe any efforts to address potential sources of bias | - | Epidemiological data. |
| Study size | 10 | Explain how the study size was arrived at | - | All patients that signed informed consent and donated blood were included. |

Continued on next page

| Quantitative variables | 11 | Explain how quantitative variables were handled in the analyses. If applicable, describe which groupings were chosen and why | - | No quantitative variables were used. |
| --- | --- | --- | --- | --- |
| Statistical methods | 12 | (*a*) Describe all statistical methods, including those used to control for confounding | 8 | Pearson’s χ^2^ test was used to verify the Hardy-Weinberg equilibrium. Differences in allele frequencies were determined using the two-sided Fisher’s exact test (GraphPad Prism Software, San Diego, USA). A p-value of p < 0.05 was considered significant. |
|  |  | (*b*) Describe any methods used to examine subgroups and interactions | - | N/A |
|  |  | (*c*) Explain how missing data were addressed | - | N/A |
|  |  | (*d*) *Cohort study*—If applicable, explain how loss to follow-up was addressed  *Case-control study*—If applicable, explain how matching of cases and controls was addressed  *Cross-sectional study*—If applicable, describe analytical methods taking account of sampling strategy | - | N/A; cases were not matched with controls. |
|  |  | (*e*) Describe any sensitivity analyses | - | N/A |
| Results | | | | |
| Participants | 13* | (a) Report numbers of individuals at each stage of study—eg numbers potentially eligible, examined for eligibility, confirmed eligible, included in the study, completing follow-up, and analysed | 9 | Exactly 215 Sudanese subjects were included in the study and were genotyped. Of these, 112 subjects had active mycetoma disease and were classified as patients, and 103 subjects were healthy controls. |
|  |  | (b) Give reasons for non-participation at each stage | - | N/A |
|  |  | (c) Consider use of a flow diagram | - | N/A |
| Descriptive data | 14* | (a) Give characteristics of study participants (eg demographic, clinical, social) and information on exposures and potential confounders | 18 | See Table 1, end of this document |
|  |  | (b) Indicate number of participants with missing data for each variable of interest | - | N/A |
|  |  | (c) *Cohort study*—Summarise follow-up time (eg, average and total amount) | - | N/A |
| Outcome data | 15* | *Cohort study*—Report numbers of outcome events or summary measures over time | - | N/A |
|  |  | *Case-control study—*Report numbers in each exposure category, or summary measures of exposure | - | See Table 1, end of this document |
|  |  | *Cross-sectional study—*Report numbers of outcome events or summary measures | - | N/A |
| Main results | 16 | (*a*) Give unadjusted estimates and, if applicable, confounder-adjusted estimates and their precision (eg, 95% confidence interval). Make clear which confounders were adjusted for and why they were included | 20 | See Table 3, end of this document. |
|  |  | (*b*) Report category boundaries when continuous variables were categorized | - | N/A |
|  |  | (*c*) If relevant, consider translating estimates of relative risk into absolute risk for a meaningful time period | - | N/A |

Continued on next page

| Other analyses | 17 | Report other analyses done—eg analyses of subgroups and interactions, and sensitivity analyses | - | N/A |
| --- | --- | --- | --- | --- |
| Discussion | | | | |
| Key results | 18 | Summarise key results with reference to study objectives | 10-11  11 | In this paper, we first showed that chitin is present in the *M. mycetomatis* grain and that two human chitinases are found in the vicinity of this grain, in reaction to exposure to *M. mycetomatis* mycetoma. Both AMCase and chitotriosidase seemed to concentrate on the chitin-containing fungal hyphae, a phenomenon that was previously found in rats infected with *Aspergillus fumigatus* [2].  Next to demonstrating that AMCase and chitotriosidase were present at the site of infection, we also provided evidence that a polymorphism in the gene for chitotriosidase, resulting in impaired enzyme activity, significantly increased the risk for the development of eumycetoma. |
| Limitations | 19 | Discuss limitations of the study, taking into account sources of potential bias or imprecision. Discuss both direction and magnitude of any potential bias | 11 | A large part of the study is *in vitro* work in which there is no bias. The main imprecision is found in the question whether the *in vitro* results can be extrapolated into the *in vivo* situation, which is emphasized by the following phrase.  However, it should be kept in mind that *in vitro* grown fungi could express different proteins than could be expressed in a grain. |
| Interpretation | 20 | Give a cautious overall interpretation of results considering objectives, limitations, multiplicity of analyses, results from similar studies, and other relevant evidence |  | *Madurella mycetomatis* is the most prevalent causative agent of eumycetoma worldwide and in Sudan in particular [1]. Many inhabitants of Sudan are exposed to this causative agent, however few of them develop mycetoma. Currently, the predisposing factors for mycetoma are not known, but some genetic polymorphisms have been associated with the development of mycetoma [4, 6, 7].  In this paper, we first showed that chitin is present in the *M. mycetomatis* grain and that two human chitinases are found in the vicinity of this grain, in reaction to exposure to *M. mycetomatis* mycetoma. Both AMCase and chitotriosidase seemed to concentrate on the chitin-containing fungal hyphae, a phenomenon that was previously found in rats infected with *Aspergillus fumigatus* [2]. Since in both mycetoma and in *A. fumigatus* infected tissue AMCase and chitotriosidase concentrated on chitin-containing fungal hyphae, it needed to be determined if this was not due to cross-reaction of fungal proteins with the antibodies used. Staining *in vitro* grown *M. mycetomatis* hyphae with an antibody for chitotriosidase showed no staining on the hyphae. Also, no staining of *A. fumigatus* hyphae occurred [2], making it likely that the chitotriosidase antibodies were specifically directed against mammalian chitotriosidase and that fungi expressed no proteins which share epitopes with this enzyme. However, it should be kept in mind that *in vitro* grown fungi could express different proteins than could be expressed in a grain. In contrast to the specificity of the chitotriosidase antibody, the AMCase antibody seemed less specific. When stained with the AMCase antibody, some staining of the *in vitro* grown *M. mycetomatis* hyphae was noted. This was not the case for *A. fumigatus* hyphae [2]. Apparently, a protein with an epitope similar to that of AMCase is located on some fungal hyphae when grown *in vitro*. Therefore the AMCase stained in the tissue samples of the patients could be the result of both expressed human AMCase and a protein of fungal origin. Since the staining was more intense in the tissue sections, we feel that it is likely that AMCase was indeed present.  Next to demonstrating that AMCase and chitotriosidase were present at the site of infection, we also provided evidence that a polymorphism in the gene for chitotriosidase, resulting in impaired enzyme activity, significantly increased the risk for the development of eumycetoma. Increased or decreased activity of the alternative human chitinase, AMCase, did not have a significant influence on the risk for eumycetoma. In *M. mycetomatis* mycetoma, chitotriosidase is apparently more crucial than AMCase. Although chitotriosidase and AMCase are both chitinases, the cleavage site of both chitinases differs. Chitotriosidase is an exochitinase, whereas AMCase is an endochitinase, referring to the site where the enzyme cleaves the chitin chain [22]. Apparently, exochitinase activity is more important in the prevention of mycetoma than endochitinase activity. This is supported by the fact that in certain diseases an association was found with only one chitinase and not with both. In genetic association studies conducted in patients with bronchial asthma, an association was reported with only one chitinase, and not with both of them [21, 23, 24], indicating that both chitinases may have the same substrate, but have a distinct function in humans. Chitin and the produced chitotriosidase seem to be important in the development of the mycetoma grain. Since many mycetoma patients have the wild type chitinases associated with normal levels and activity of these enzymes, however, polymorphisms in these enzyme-encoding genes are clearly not the only factors that determine the risk for *M. mycetomatis* mycetoma.  Many previous reports showed that polarization of the immune response seems to play a role in the development of mycetoma [4, 7, 25-28]. The development of mycetoma is associated with a Th2 response. Based on immunohistochemistry studies in various mycetoma causative agents, it appeared that the cytokine pattern surrounding the mycetoma grain is a Th2 response. IL-10 and IL-4, both Th2-associated cytokines, were highly expressed around the fungal grain [7, 26, 27]. A different study showed that after stimulation of peripheral blood mononuclear cells (PBMCs) with mycetoma antigens, a Th2 response developed in mycetoma patients and a Th1 response developed in healthy endemic controls [25]. Indirect evidence for a Th2 response was also found in the association between schistosomiasis, associated with a Th2 response, and eumycetoma [5]. Not only cytokines, but also other mediators matching Th2 response were reported in mycetoma.  Sandler *et al* [28] showed that mice with a Th2 response have increased expression of matrix metalloproteinases (MMPs) and of tissue inhibitor of MMP-1 (TIMP-1). Furthermore, AMCase was induced in Th2-polarized mice [28], which was confirmed by several other studies [29-31]. Furthermore, Geneuglijk *et al* confirmed that MMP-2 and MMP-9 were expressed in the mycetoma lesion [8]. In this paper we also demonstrated that AMCase and chitotriosidase are expressed in the mycetoma lesion.  In contrast to AMCase, which is induced in a Th2 response, chitotriosidase is produced in the environment of a Th1 response [32]. In our study we showed that impaired function of chitotriosidase increases the risk to develop mycetoma.  Since Elagab et al already demonstrated that the PBMCs of healthy endemic controls produce Th1 cytokines when exposed to *M. mycetomatis* antigens, it is likely that they also produce high levels of chitotriosidase in order to eliminate *M. mycetomatis*. In individuals with a genotype resulting in impaired chitotriosidase activity, elimination of *M. mycetomatis* could be less efficient, leading to the development of a mycetoma lesion. More research is needed to unravel the exact role of the host in the development of the mycetoma grain. |
| Generalisability | 21 | Discuss the generalisability (external validity) of the study results | 14 | In this study we demonstrated that the grain caused by *Madurella mycetomatis*, contains chitin. The human immune system produced both AMCase and chitotriosidase in the vicinity of this grain. Only the 24-bp insertion in the gene for chitotriosidase was associated with the development of mycetoma caused by *M. mycetomatis*. |
| Other information | |  | | |
| Funding | 22 | Give the source of funding and the role of the funders for the present study and, if applicable, for the original study on which the present article is based | - | Funding information is stated in the funding disclosure section in the online submission system. |

*Give information separately for cases and controls in case-control studies and, if applicable, for exposed and unexposed groups in cohort and cross-sectional studies.

**Note:** An Explanation and Elaboration article discusses each checklist item and gives methodological background and published examples of transparent reporting. The STROBE checklist is best used in conjunction with this article (freely available on the Web sites of PLoS Medicine at http://www.plosmedicine.org/, Annals of Internal Medicine at http://www.annals.org/, and Epidemiology at http://www.epidem.com/). Information on the STROBE Initiative is available at [www.strobe-statement.org](http://www.strobe-statement.org).

Table 1.

| **Characteristic** |  | **Mycetoma**  **patients  (n=112)** | **Endemic**  **controls**  **(n=103)** |
| --- | --- | --- | --- |
| **Gender (male/female)** |  | 79/33 | 77/26 |
| **Mean duration in years (range)** |  | 6.9  (1-27) | N/A |
| **Mycetoma lesion site*** | Foot | 87 | N/A |
|  | Hand | 12 | N/A |
|  | Lower leg | 14 | N/A |
| **Mycetoma lesion size** | Small (<5 cm) | 55 | N/A |
|  | Moderate (5-10 cm) | 20 | N/A |
|  | Massive (>10 cm) | 38 | N/A |

Table 3.

| **Gene Polymorphism** | **Genotype** | **Enzyme activity*** | **Patients (%)**  **n=112** | **Controls (%)**  **n=103** | **HWE****  **p-value** | **p-value** | **Odds ratio (95% CI interval)** |
| --- | --- | --- | --- | --- | --- | --- | --- |
| Chitotriosidase 24-bp insertion | Wildtype | Normal | 84 (75%) | 94 (91%) | 0.106 | 0.004 | 2.9 (1.4-6.1) |
|  | Heterozygous 24-bp insertion | Decreased | 27 (24%) | 8 (8%) |  |  |  |
|  | Homozygous 24-bp insertion | Impaired | 1 (1%) | 1 (1%) |  |  |  |
| AMCase A50G | AA | Normal | 92 (82%) | 83 (81%) | 0.940 | 0.647 | 1.1 (0.7-1.8) |
|  | AG | Normal | 14 (13%) | 19 (18%) |  |  |  |
|  | GG | Decreased | 6 (5%) | 1 (1%) |  |  |  |
| AMCase A290G | AA | Normal | 74 (66%) | 67 (65%) | 0.657 | 0.717 | 1.2 (0.6-2.1) |
|  | AG | Normal | 30 (27%) | 33 (32%) |  |  |  |
|  | GG | Increased | 8 (7%) | 3 (3%) |  |  |  |
| AMCase 10-bp insertion 5’UTR | Wildtype | Normal | 73 (65%) | 66 (64%) | 0.578 | 0.720 | 1.1 (0.7-1.8) |
|  | Heterozygous 10-bp insertion | Normal | 31 (28%) | 34 (33%) |  |  |  |
|  | Homozygous 10-bp insertion | Increased | 8 (7%) | 3 (3%) |  |  |  |
